# Association of *ARHGAP*18 polymorphisms with schizophrenia in the Chinese-Han population

**DOI:** 10.1371/journal.pone.0175209

**Published:** 2017-04-06

**Authors:** Weiyun Guo, Yaqi Cai, Hongxing Zhang, Yongfeng Yang, Ge Yang, Xiujuan Wang, Jingyuan Zhao, Juntang Lin, Jinfu Zhu, Wenqiang Li, Luxian Lv

**Affiliations:** 1 College of Life Science and Technology, Xinxiang Medical University, Xinxiang, China; 2 Henan Key Lab of Biological Psychiatry, Xinxiang Medical University, Xinxiang, China; 3 Department of Psychiatry, Henan Mental Hospital, The Second Affiliated Hospital of Xinxiang Medical University, Xinxiang, China; 4 Institute of Anatomy I, Friedrich Schiller University Jena, Jena, Germany; 5 Department of Psychology, Xinxiang Medical University, Xinxiang, China; University of Texas Health Science Center at San Antonio Cancer Therapy and Research Center at Houston, UNITED STATES

## Abstract

Numerous developmental genes have been linked to schizophrenia (SZ) by case-control and genome-wide association studies, suggesting that neurodevelopmental disturbances are major pathogenic mechanisms. However, no neurodevelopmental deficit has been definitively linked to SZ occurrence, likely due to disease heterogeneity and the differential effects of various gene variants across ethnicities. Hence, it is critical to examine linkages in specific ethnic populations, such as Han Chinese. The newly identified RhoGAP ARHGAP18 is likely involved in neurodevelopment through regulation of RhoA/C. Here we describe four single nucleotide polymorphisms (SNPs) in ARHGAP18 associated with SZ across a cohort of >2000 cases and controls from the Han population. Two SNPs, rs7758025 and rs9483050, displayed significant differences between case and control groups both in genotype (*P* = 0.0002 and *P* = 7.54×10^−6^) and allelic frequencies (*P* = 4.36×10^−5^ and *P* = 5.98×10^−7^), respectively. The AG haplotype in rs7758025−rs9385502 was strongly associated with the occurrence of SZ (*P* = 0.0012, *OR* = 0.67, 95% *CI* = 0.48–0.93), an association that still held following a 1000-times random permutation test (*P* = 0.022). In an independently collected validation cohort, rs9483050 was the SNP most strongly associated with SZ. In addition, the allelic frequencies of rs12197901 remained associated with SZ in the combined cohort (*P* = 0.021), although not in the validation cohort alone (*P* = 0.251). Collectively, our data suggest the *ARHGAP18* may confer vulnerability to SZ in the Chinese Han population, providing additional evidence for the involvement of neurodevelopmental dysfunction in the pathogenesis of schizophrenia.

## Introduction

Schizophrenia (SZ) is among the most severe and difficult to treat psychiatric disorders due to variable expression of psychotic symptoms, mood deregulation, and cognitive dysfunction[1]. There is also considerable heterogeneity in disease heritability, implying that SZ arises from a complex interaction of multiple genetic susceptibility factors; thus, there is no unified pathogenic model [2]. Compelling evidence points to disturbances in neurodevelopment during the prenatal and early postnatal periods that impact brain maturation during adolescence and early adulthood, ultimately leading to the delayed emergence of psychiatric symptoms[3]. It is thus believed that different allelic combinations of neurodevelopmental genes (haplotypes) may predispose individuals to SZ and other major psychiatric disorders [4, 5].

Neurodevelopmental abnormalities may result from prenatal immune activation [6], alimentary deficiency [7], and genetic factors. Indeed, multiple genetic factors have been linked to schizophrenia susceptibility[8–10], including genes associated with Rho GTPase signaling pathways[11]. Rho GTPase activating proteins (RhoGAPs) are a large protein family containing approximate 80 members that stimulate GTP hydrolysis, thereby turning the GTP-bound active form of Rho into a inactive GDP-bound form[12, 13]. The Rho family GTPase play a critical role in many aspects of neuronal development, including neurite outgrowth[14, 15], neuronal differentiation [14], axon guidance[15–17], and synaptic formation and maintenance[18,19]. Likewise, many RhoGAP proteins have been linked to neurodevelopmental processes and related disabilities. For example, oligophrenin-1 encodes a RhoGAP involved in X-linked mental retardation [20, 21]. Recently, dysfunction of RhoGAPX-1 and ARHGAP6 has been implicated in a wide range of developmental defects seen in microphthalmia with linear skin defects syndrome[22, 23], while srGAP1, srGAP2, and srGAP3 have been linked to mental retardation, schizophrenia, and seizures[24]. Considering the functional redundancy of many RhoGAP proteins, these findings suggest that additional family members are also involved in pathological conditions related to aberrant neurodevelopment.

ARHGAP18 is a newly identified RhoGAP capable of regulating RhoA and RhoC activities in a cell type-specific context [25, 26]. Although the functions of ARHGAP18 in the central nervous system are presently unknown, recent studies have shown a potential correlation between genetic polymorphisms of ARHGAP18 and the occurrence of schizophrenia. Through the combinatorial use of a genome-wide screening and neuroimaging, single nucleotide polymorphisms (SNPs) within *ARHGAP18* were associated with schizophrenia [27, 28]. However, these studies were based on Western populations and not validated in an independent case−control study. Herein, we evaluated the association of *ARHGAP18* polymorphisms and schizophrenia in a large Chinese Han population of SZ patients and matched controls.

## Materials and methods

### Subjects

All participants were recruited from northern Henan Province and had four biological grandparents of Han Chinese ancestry. The Structured Clinical Interview for Diagnostic and Statistical Manual of Mental Disorders-Fourth Edition IV (DSM-IV) (1994) Axis I Disorders was used to exclude individuals with a history of severe medical complications (such as diabetes, cardiovascular disease, hypertension), organic brain diseases, concomitant major psychiatric disorders, and/or substance dependence. The discovery cohort consisted of 528 patients (264 males and 264 females; mean age: 27.32 ± 8.03 years old) and 528 healthy controls matched for sex ratio, age, and ethnicity (264 males and 264 females; mean age: 27.73 ± 8.01 years old). The validation cohort consisted of 860 patients (430 males and 430 females; mean age: 28.34 ± 9.25 years old) and 860 healthy matched controls (430 males and 430 females; mean age: 29.58 ± 7.29 years old). For each patient, the diagnosis of SZ was confirmed by at least two psychiatrists according to the DSM-IV criteria for paranoid SZ. All healthy volunteers were recruited from Xinxiang Medical University, Xinxiang city, and surrounding communities and villages by posters in Physical Examination Center and hospitals in towns and counties. Any individual with a personal or family history of mental or neurological diseases was excluded. The controls were well matched to the patient group for gender ratio (1:1 for both groups), age (*F* = 0.621, *P* = 0.464), and ethnicity (all unrelated, living in North Henan Province, and with all biological grandparents of Chinese-Han ancestry).

Written informed consent was obtained for all participants. The study was approved by the ethics committee of the Second Affiliated Hospital of Xinxiang Medical University.

### Genotyping

A peripheral blood sample was drawn from each subject into vacutainer tubes containing the anticoagulant ethylenediaminetetraacetic acid. Genomic DNA was extracted from leukocytes using the RelaxGene Blood DNA System (Tiangen Biotech., Beijing, China). In the discovery stage, the genotypes of 35 SNPs in *ARHGAP18* were evaluated using the Illumina GoldenGate assay on a BeadStation 500G Genotyping System (Illumina, Inc., San Diego, CA, USA) according to the manufacturer’s instructions.

Validation of specific SNPs, including rs9483050, rs7758025, rs12197901, and rs9492347, was performed using the TaqMan genotyping method according to the manufacturer’s protocol, with allelic discrimination and analysis performed on an ABI Prism 7900 Sequence Detection System (Applied Biosystems, Foster City, CA, USA). The ABI Taqman probe sequences are listed in S1 Table. To evaluate the quality of genotyping, 5% of the samples were randomly selected and re-genotyped. The genotyping consistency rate was more than 98%.

### Bioinformatics analyses

All genotype data were examined for cluster separation using Illumina quality scores generated by the software. Poorly performing SNPs as defined by a GenTrain score < 0.4 or a cluster separation score < 0.6 were excluded. SNPs were further excluded if controls were not in Hardy—Weinberg equilibrium. As a genotyping quality control, four SNPs were genotyped in duplicate in 100 samples by DNA sequencing.

Genotypes and allele frequencies in SZ and control subjects were compared using the Haploview V4.1 program with Bonferroni correction to exclude type I errors (including from other SNPs in the same GoldenGate 384 assay relevant to a different experimental design). Hardy—Weinberg equilibrium was also evaluated using this program,. The standardized measures of linkage disequilibrium (LD) coefficients (D′), haplotype frequency, haplotype block, and haplotype association were assessed using Haploview V4.1.

Allele and genotype counts were compared by the Pearson chi-square test. A power analysis was performed using the Genetic Power Calculator[29]. Genotyping data (include other SNPs in the same GoldenGate 384 assay relevant to a different experimental design) were analyzed using the Markov chain Monte Carlo algorithm in Structure 2.3 [30] to generate population stratification assignments for all individuals. Odds ratios (ORs) and 95% confidence intervals (95% CIs) were calculated to evaluate the effect of different alleles and haplotypes on SZ risk. The haplotype frequencies were estimated using the expectation maximization (EM) algorithm.

## Results

We selected a total of thirty-five SNPs in *ARHGAP18* for genotypic distribution analysis in 528 patients with schizophrenia and 528 healthy controls. All SNPs evaluated demonstrated a minor allele frequency greater than 5% in the studied samples. Power analysis revealed that the total sample size (n = 1056) had the power (0.86) to detect a small (r = 0.1–0.23) effects and the power (1.00) to detect both medium(r = 0.24–0.36) and large(r > 0.37) effects on genotype distributions. The genotype and allele frequencies of these SNPs in patients and controls are shown in Table 1.

**Table 1:** Genotype and allele frequencies of thirty-two SNPs in the ARHGAP18 gene of schizophrenia patients and controls.

SNP#	dbSNP ID	Allele(D/d)^a^	Patients						Controls						*P*-value	
			n^b^	HWE(*P*)	Genotype			MAF	n^b^	HWE(*P*)	Genotype			MAF		
					DD	Dd	dd				DD	Dd	dd		Genotype	Allele
1	rs6569610	A/T	527	0.931	304	193	30	0.240	527	0.109	293	209	25	0.246	0.524	0.761
2	rs9492347	A/G	528	<0.001	480	41	7	0.052	528	0.821	460	66	2	0.066	**0.011**	0.167
3	rs6923483	A/G	527	0.451	262	214	51	0.300	527	0.646	279	206	42	0.275	0.458	0.211
4	rs4895852	G/A	527	0.719	178	260	89	0.416	527	0.908	163	259	105	0.445	0.371	0.172
5	rs7758025	G/A	528	0.041	417	99	12	0.116	528	0.213	463	61	4	0.065	**0.0002**	**4.36×10**^**−5**^
6	rs9385502	G/A	527	0.203	277	218	32	0.268	527	0.305	270	221	36	0.278	0.841	0.591
7	rs9402145	A/G	528	0.445	390	130	8	0.138	528	0.708	388	128	12	0.144	0.663	0.708
8	rs3813366	A/G	527	0.950	139	264	124	0.486	527	0.436	164	252	111	0.450	0.216	0.097
9	rs17057516	A/G	514	<0.001	383	94	37	0.163	512	<0.001	375	106	31	0.164	0.514	0.969
10	rs9483048	A/T	525	0.970	207	245	73	0.372	528	0.671	218	246	64	0.354	0.647	0.385
11	rs11753915	C/A	528	0.852	359	152	17	0.176	528	0.364	338	173	17	0.196	0.370	0.240
12	rs9483050	A/G	528	0.194	379	132	17	0.157	528	0.551	442	81	5	0.086	**7.54×10**^**−6**^	**5.98×10**^**−7**^
13	rs12216321	A/G	525	<0.001	429	72	24	0.114	525	<0.001	428	81	16	0.108	0.344	0.627
14	rs13193932	C/G	528	<0.001	97	388	43	0.449	528	<0.001	80	417	31	0.454	0.099	0.827
15	rs12197901	A/G	528	0.339	364	145	19	0.173	528	0.452	395	121	12	0.137	0.082	**0.022**
16	rs9388722	A/G	527	0.336	158	251	118	0.462	528	0.296	155	273	100	0.448	0.296	0.514
17	rs10499164	G/A	528	0.011	456	65	7	0.075	528	0.447	445	78	5	0.083	0.438	0.468
18	rs9388723	A/G	527	0.889	288	204	35	0.260	528	0.169	262	229	37	0.287	0.256	0.164
19	rs7765511	A/G	528	0.378	489	39	0	0.037	526	0.924	479	46	1	0.046	0.433	0.316
20	rs12202304	C/A	526	0.995	481	44	1	0.044	528	0.224	475	53	0	0.050	0.393	0.483
21	rs6928167	G/A	527	0.050	160	240	127	0.469	528	0.427	166	252	110	0.447	0.445	0.317
22	rs11968342	G/C	528	0.165	418	107	3	0.107	527	0.863	409	111	7	0.119	0.413	0.400
23	rs10872345	A/G	528	0.507	236	239	53	0.327	528	0.325	251	233	44	0.304	0.503	0.261
24	rs9398917	G/A	528	0.832	323	181	24	0.217	528	0.385	348	165	15	0.185	0.153	0.064
25	rs1476042	C/A	528	0.160	175	244	109	0.438	528	0.524	185	261	82	0.402	0.097	0.103
26	rs11962358	A/G	527	0.417	211	238	78	0.374	527	0.783	191	250	86	0.400	0.432	0.211
27	rs17467757	G/A	527	0.452	400	116	11	0.131	528	0.481	400	117	11	0.132	0.998	0.962
28	rs763132	A/C	527	0.783	191	250	86	0.400	528	0.143	189	240	99	0.415	0.569	0.501
29	rs17057659	G/A	527	0.408	285	200	42	0.269	528	0.396	276	206	46	0.282	0.813	0.512
30	rs6917887	G/A	527	0.924	183	256	88	0.410	528	0.106	173	274	81	0.413	0.554	0.888
31	rs11154495	C/A	527	0.274	242	222	63	0.330	528	0.647	241	228	59	0.328	0.899	0.902
32	rs3752536	G/A	521	0.052	324	164	33	0.221	525	0.859	325	177	23	0.212	0.322	0.643
33	rs17057685	C/A	528	0.323	291	196	41	0.263	528	0.453	272	209	47	0.287	0.480	0.223
34	rs11154496	G/A	528	0.425	446	80	2	0.080	528	0.020	453	68	7	0.078	0.149	0.871
35	rs9402163	C/G	527	0.231	351	153	23	0.189	528	0.676	368	144	16	0.167	0.381	0.183

^a^ Major/minor allele, major and minor alleles are denoted by D and d, respectively.

^b^ Number of samples which are well genotyped.

^c^ the significance of bold values is p<0.05.

The genotype and allelic frequencies of two SNPs, rs7758025 and rs9483050 displayed significant differences between the case and control groups (rs7758025: genotype *P* = 0.0002; allele *P* = 4.36×10^-5^; rs9483050: genotype *P* = 7.54×10^-6^; allele *P* = 5.98×10^-7^). In addition, rs9492347 genotype frequency was associated with schizophrenia (*P* = 0.011) as was rs12197901 allelic frequency (*P* = 0.022). The genotypic distribution of these four SNPs did not demonstrate significant deviations from Hardy−Weinberg equilibrium in the control group.

**Fig 1:**
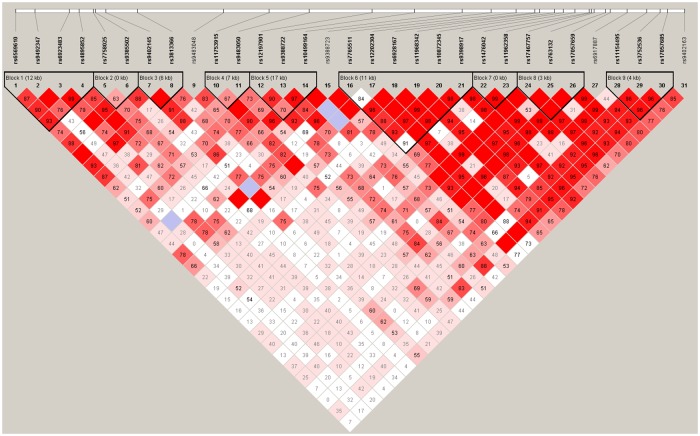
LD structure and the D′ values for the 31 Single Nucleotide Polymorphisms (SNPs).

We next performed LD analysis using pairs of SNPs to further analyze the haplotype structure. As shown in Fig 1, Table 2, the LD plot consisted of thrity-five SNPs. Haplotypes GG and GA in the LD block rs7758025−rs9385502 showed minimal difference between the case and control groups (*P* = 0.175 and *P* = 0.232, respectively), while haplotype AG was strongly associated with schizophrenia (*P* = 0.0012, *OR* = 0.67, 95% *CI* = 0.48−0.93). These associations remained following a 1000-times random permutation test (*P* = 0.022). Haplotype CG in the LD block rs11753915−rs9483050 was also associated with schizophrenia (*P* = 9.6×10^−6^, *OR* = 0.58, 95% *CI* = 0.44−0.78) even after Bonferroni correction (*P* = 0.0001).

**Table 2:** Haplotype analysis among SZ and controls.

Haplotype^a^	Haplotype frequencies^b^		χ^2^	*P*-value^c^	OR (95% CI)
	Patients	Controls			
rs7758025–rs9385502					
GG	666.6(63.1)	696.4(65.9)	1.837	0.175	1.00
GA	266.4(25.2)	290.6 (27.5)	1.429	0.232	1.06 (0.86–1.30)
AG	106.8(10.1)	66.0(6.3)	10.489	0.0012(0.022)	0.67 (0.48–0.93)
rs11753915–rs9483050					
CA	716.8(67.9)	760.6(72.0)	4.323	0.038	1.00
AA	173.2(16.4)	204.4(19.4)	3.138	0.077	1.12 (0.88–1.41)
CG	153.2(14.5)	88.4(8.4)	19.628	9.6×10^−6^(0.0001)	0.58 (0.44–0.78)

^a^ Haplotypes were omitted from analysis if the estimated haplotype probabilities were less than 5%.

^b^ Frequencies are shown in parenthesis (%).

^c^ *P* values in the parenthesis were analyzed with 1000 random permutations, Global haplotype association *P*-value all <0.0001.

We then re-tested the four *ARHGAP18* SNPs associated with schizophrenia in an independent validation cohort of 860 patients and 860 controls. In stage 2, the ample size gets a high power (0.93). As shown in Table 3, both genotype and allelic frequencies of rs7758025 and rs9483050 SNPs displayed strong associations with schizophrenia in the validation cohort. Of note, rs9483050 appeared most strongly associated with the disease state across the two cohorts. In addition, the allelic frequency of rs12197901 remained associated with schizophrenia in the combined analysis (*P* = 0.021), although not in the validation cohort alone (*P* = 0.251). The trends observed for the association of rs9492347 with SZ were not confirmed in the validation cohort or combined analysis.

**Table 3:** SNP association analysis for ARHGAP18 in stage 2 and combined sample set.

Stage	dbSNP ID	Allele(D/d)^a^	Patients						Controls						*P*-value	
			n^b^	HWE(*P*)	Genotype			MAF	n^b^	HWE(*P*)	Genotype			MAF		
					DD	Dd	dd				DD	Dd	dd		Genotype	Allele
1	rs9492347	A/G	528	<0.001	480	41	7	0.052	528	0.821	460	66	2	0.066	0.011	0.167
2			855	0.001	733	110	12	0.078	860	0.009	742	108	10	0.074	0.886	0.664
1&2			1383	<0.001	1213	151	19	0.068	1388	0.045	1202	174	12	0.071	0.196	0.662
1	rs7758025	G/A	528	0.041	417	99	12	0.116	528	0.213	463	61	4	0.065	2.00×10^−4^	4.36×10^−5^
2			860	0.006	679	161	20	0.117	860	0.935	714	139	7	0.089	0.013	0.007
1&2			1388	<0.001	1096	260	32	0.117	1388	0.439	1177	200	11	0.080	2.79×10^−5^	4.28×10^−6^
1	rs9483050	A/G	528	0.194	379	132	17	0.157	528	0.551	442	81	5	0.086	7.54×10^−6^	5.98×10^−7^
2			860	0.002	646	186	28	0.141	860	0.529	709	142	9	0.093	9.20×10^−5^	1.35×10^−5^
1&2			1388	0.001	1025	318	45	0.147	1388	0.387	1151	223	14	0.090	1.80×10^−9^	7.28×10^−11^
1	rs12197901	A/G	528	0.339	364	145	19	0.173	528	0.452	395	121	12	0.137	0.082	0.022
2			860	0.217	593	236	31	0.173	860	0.67	607	233	20	0.159	0.845	0.251
1&2			1388	0.119	957	381	50	0.173	1388	0.911	1002	354	32	0.151	0.050	0.021

^a^ Major/minor allele, major and minor alleles are denoted by D and d, respectively.

^b^ Number of samples which are well genotyped.

## Discussion

Herein, we describe associations between *ARHGAP18* polymorphisms and schizophrenia in a Chinese Han population. In stage one, we screened SNPs in *ARHGAP18* from GWAS data, and discovered four SNPs, rs7758025, rs9483050, rs9492347 and rs12197901, associated with schizophrenia. In stage two, we validated our findings in an independent cohort and demonstrated that rs9483050 is strongly associated with schizophrenia. Our data suggest that allelic variation in the *ARHGAP18* gene may confer vulnerability to SZ in the Chinese Han population, providing additional evidence for the involvement of disrupted neurodevelopmental signals in disease pathogenesis.

Although the detailed molecular events during SZ progression remain elusive, it is widely accepted that abnormalities in early brain development caused by inherited genetic variants alter critical developmental and maturational processes, resulting in eventual emergence of disabling psychoses[31–33]. A plethora of genes have polymorphisms associated with SZ [9, 34–38]. Nevertheless, due to complexity of epidemiology, including ethnicity and disease subtype, our understanding of disease heritability remains limited and it is likely that many more SZ susceptibility genes remain to be identified.

*ARHGAP18* (6q22.33) lies within a previously reported SZ candidate region (SCR) identified by Lerer et al [39], in an ethnically homogeneous family-based Arab—Israeli sample [27, 28]. There are 134 genes in this risk region, of which several contain SNPs enriched in sporadic SZ cases, such as dystrobrevin-binding protein 1 (DTNBP1) and laminin alpha-2 (LAMA2). Given the large genomic distance spanned and the difference in localization of linkage peaks, it is possible that this region harbors additional SZ susceptibility genes, one of which may be *ARHGAP18*. In fact, there are several reports on the involvement of *ARHGAP18* SNPs in human diseases, including SZ. Recurrent chromosomal imbalances affecting the *ARHGAP18* locus were observed in six of nine patients with neurofibromatosis type 1[40]. Also, Potkin *et al*. used brain activation pattern during a working memory task as a quantitative trait to interpret GWAS data and identified *ARHGAP18* as a SZ risk gene [27]. Specifically, they found six SNPs within *ARHGAP18*, rs12664247, rs4509146, rs11154490, rs2051632, rs17469847 and rs10484284, with statistically significant relationships in both the discovery and validation cohorts.

Herein, we used large discovery and validation cohorts to identify the rs9492347, rs7758025, and rs9483050 SNPs of *ARHGAP18* as susceptibility loci for SZ. Our results are intriguing in several ways. First, these SNPs were not associated with SZ in GWASs interrogating *ARHGAP18*, although those studies did not use the sample SNP panel studied here. Also, the number of included samples in our study is much higher than previous studies on *ARHGAP18* SNPs. Moreover, our study extends the risk of SZ conferred by *ARHGAP18* SNPs from Western populations to the Eastern Han population.

*ARHGAP18* encodes one of approximately 80 RhoGAP proteins [12, 13]. As a RhoGAP, ARHGAP18 mainly serves as a molecular switch for controlling the balance between active and inactive Rho proteins to regulate Rho-mediated signaling pathways. Rho GTPases, a protein family composed of 22 members in mammals, are known as important modulators of the actin cytoskeleton influencing neuronal morphology and migration [41, 42]. In addition, Rho GTPases are also reportedly involved in the regulation of growth factor-linked signal pathways.

Among members of the Rho GTPase family, RhoA is the main molecule responding to ARHGAP18 regulation. ARHGAP18-knockdown cells demonstrated impaired cell spreading, premature formation of stress fibers, and sustained activation of RhoA upon cell attachment, resulting in inhibition of cell migration [25, 26]. Although the neurodevelopmental function of ARHGAP18 has not been elucidated, numerous studies have suggested a critical role for RhoA in neurogenesis and maturation [43–46]. Abolishing RhoA activity in the postnatal stage led to major changes in density and absolute number of neurons in the somatosensory cortex [47]. Also, deletion of RhoA from neural progenitor cells in mice resulted in abnormal locomotor behavior [48, 49]. Of note, all three identified SNPs are located in the intron region, which is common for most top hit SZ-associated SNPs revealed by GWASs. The presence of these intronic SNPs may regulate ARHGAP18 mRNA splicing in a trans-acting manner, thereby leading to malfunction of the ARHGAP18−RhoA axis in neurodevelopment.

## Conclusion

In summary, our study provides novel data suggesting an association between ARHGAP18 and SZ susceptibility. Replication studies in different ethnic populations, particularly in patients with defined SZ phenotypes, and more samples, are required to confirm the role of ARHGAP18 variants in SZ.

## Supporting information

S1 Table Taqman probe sequences of four SNPs. (DOC)
