# Supplementary material for: Association of ARHGAP18 polymorphisms with schizophrenia in the Chinese-Han population
Source: PLoS One. 2017 Apr 6;12(4):e0175209. doi: 10.1371/journal.pone.0175209 (PMC5383423; doi:10.1371/journal.pone.0175209)
Supplement: S1 Table — (DOC) [file pone.0175209.s001.doc]

S1 Table Taqman probe sequences of four SNPs

| SNP ID | Context Sequence [VIC/FAM] |
| --- | --- |
| rs9483050 | CCATTATTATTTATGCTTTTAAAGT[A/G]GAACTCAGTATTGGTGTATAAAACC |
| rs7758025 | TGTAATTGCTTTGTGTGTCTATTTA[C/T]TCTCCCTAATAAAATGGCATAATTT |
| rs12197901 | AGATCTGGGATCCAAGAAAAGTATT[C/T]AAATATCATCCAGGAATTTCATTCT |
| rs9492347 | AAAGAAGCAACACGATATAAGACTG[C/T]ACAGTGAGTATGGGATTGTGGAAGA |
